# Metabolic Syndrome and Heart Transplantation: An Underestimated Risk Factor?

**DOI:** 10.3389/ti.2024.11075

**Published:** 2024-03-08

**Authors:** Sandro Sponga, Igor Vendramin, Veronica Ferrara, Michela Marinoni, Giulia Valdi, Concetta Di Nora, Chiara Nalli, Giovanni Benedetti, Daniela Piani, Andrea Lechiancole, Maria Parpinel, Uberto Bortolotti, Ugolino Livi

**Affiliations:** ^1^ Department of Medicine (DAME), University of Udine, Udine, Italy; ^2^ Cardiothoracic Department, University Hospital of Udine, Udine, Italy

**Keywords:** heart transplantation, metabolic syndrome, cardiac allograft vasculopathy, long-term mortality, long-term outcome

## Abstract

Metabolic Syndrome (MetS), a multifactorial condition that increases the risk of cardio-vascular events, is frequent in Heart-transplant (HTx) candidates and worsens with immunosuppressive therapy. The aim of the study was to analyze the impact of MetS on long-term outcome of HTx patients. Since 2007, 349 HTx patients were enrolled. MetS was diagnosed if patients met revised NCEP-ATP III criteria before HTx, at 1, 5 and 10 years of follow-up. MetS was present in 35% of patients pre-HTx and 47% at 1 year follow-up. Five-year survival in patients with both pre-HTx (65% vs. 78%, *p* < 0.01) and 1 year follow-up MetS (78% vs 89%, *p* < 0.01) was worst. At the univariate analysis, risk factors for mortality were pre-HTx MetS (HR 1.86, *p* < 0.01), hypertension (HR 2.46, *p* < 0.01), hypertriglyceridemia (HR 1.50, p=0.03), chronic renal failure (HR 2.95, *p* < 0.01), MetS and diabetes at 1 year follow-up (HR 2.00, *p* < 0.01; HR 2.02, *p* < 0.01, respectively). MetS at 1 year follow-up determined a higher risk to develop Coronary allograft vasculopathy at 5 and 10 year follow-up (25% vs 14% and 44% vs 25%, *p* < 0.01). MetS is an important risk factor for both mortality and morbidity post-HTx, suggesting the need for a strict monitoring of metabolic disorders with a careful nutritional follow-up in HTx patients.

## Graphical Abstract

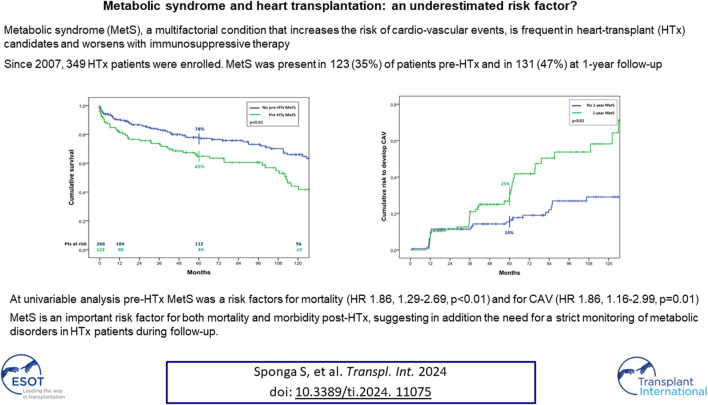

## Introduction

The Metabolic Syndrome (MetS) is a multi-factorial condition, explained as the association of several cardiovascular risk factors, including elevated glucose, hypertension, abdominal obesity and dyslipidemias that cluster in the same subject. The physiopathological process of its development is complex, but insulin resistance and abdominal obesity play a key role [1]. The prevalence of MetS in the general population varies from 18% to 39%, depending on the diagnostic criteria used, demographic, and racial differences, and this condition is correlated to an enhanced risk to develop chronic related diseases, such as cancer, neurological disorders and cardiovascular diseases [2, 3]. In particular, the presence of MetS has been associated with a twofold increase in the risk of development of cardiovascular disease, cardiovascular mortality, and nonfatal acute myocardial infarction and stroke, and a 1.5-fold increase in all-cause mortality [4].

Despite many advances in patients’ management and pharmacological treatment, MetS represents a real burden in heart transplanted (HTx) patients, mainly due to the side effects of immunosuppressive therapy, which severely affects their long-term outcomes. Moreover, the biochemical features of MetS have been strongly related to the presence and progression of the cardiac allograft vasculopathy (CAV), a peculiar complication after HTx, characterized by a diffuse intimal hyperplasia and fibrosis related to chronic rejection, but also to cardiovascular risk factors, including hypertension, diabetes or dyslipidemia [2, 5]. In literature, the prevalence of MetS in HTx patients is reported to be around 40%, but these previous studies involved a limited number of patients in limited follow-ups [4].

The aim of the present study was to assess the prevalence of MetS in HTx patients of University Hospital of Udine over 10 years of follow-up, and to evaluate the impact on the long-term outcome in terms of morbidity and mortality.

## Materials and Methods

### Study Design, Patient Population and Data Collection

From January 2007 to September 2021, 349 subjects underwent HTx at the University Hospital of Udine and were enrolled in this retrospective observational study. Data were collected from clinical informatic system and patient charts, considering 4 timepoints: before HTx surgery (baseline); at 1, 5 and 10 years of follow-up after HTx.

At baseline timepoint, demographic and clinical pre-HTx data were collected.

At the follow-up timepoints, long-term outcome and mortality, laboratory tests parameters, including a complete blood count, fasting blood glucose, lipid profile, renal function, echocardiogram exam parameters, drugs therapy, anthropometric measures and blood pressure values were collected.

The present study was approved by the local Institutional Review Board (code 17_2020) and informed consent was obtained as required by the study authorizing entity.

### Follow-Up and Immunosuppression Therapy

The postoperative and long-term follow-up protocol for HTx patients included endomyocardial biopsies made every week during the first month, every 15 days in months 2 and 3, and monthly or bimonthly up to 12 months, and if required thereafter. Coronary angiography was performed at the first year and every 2 years afterwards or on clinical requirement. Clinical follow-up was conducted by a dedicated team including a cardiac surgeon, a cardiologist, a nurse and a psychologist every 15 days during the first 3 months, every month between months 3 and 12, every 3 months between 1 and 3 years, every 4 months between 3 and 5 years and every 6 months after 6 years from transplantation [6]. At each postoperative control, right and left ventricular function and morphology were evaluated by transthoracic 2D-Echo.

During clinical evaluation, adherence to immunosuppressive treatment was also verified, and therapy modified or titrated according to case-specific conditions. The first-line immunosuppression included cyclosporine (Cys) or Tacrolimus, mycophenolate mofetil (MMF), and corticosteroids in all patients. Everolimus was administered instead of MMF in case of patients with diagnosis of CAV. All recipients received induction therapy with antithymocyte globulins, whenever possible. A standardized protocol for corticosteroid withdrawal, within 6 months after HTx, and Cys serum concentration lowering was applied guided by serial endomyocardial biopsies coupled with clinical and laboratory findings [7].

### Metabolic Syndrome Diagnosis

According to modified, revised NCEP-ATP III (Third Report of the National Cholesterol Education Program) criteria [1], diagnosis of MetS was made when the patient met at least three of the following criteria:

• Triglycerides (TGL) levels ≥150 mg/dL or drug treatment for hypertriglyceridemia
• High-density lipoprotein (HDL)-C < 40 mg/dL in men and <50 mg/dL in women or drug treatment to raise HDL-C levels
• Diabetes mellitus (DM) and treatment for elevated glucose or fasting glucose levels ≥100 mg/dL
• Blood pressure ≥130/85 mmHg or antihypertensive drug treatment
• Waist circumference >102 cm in men and >88 cm in women.

This latter parameter was substituted with body mass index (BMI) > 30 as the cut-off point for obesity. This substitution was already used also in other papers [4].

The diagnostic criteria used for MetS in this study have been used in many different studies associating MetS with cardiovascular disease in both the general population and in HTx recipients [8, 9].

### Definitions

Cardiac allograft vasculopathy (CAV) was diagnosed by angiography and defined according to the ISHLT classification [10]. Infections were registered as any episodes requiring antibiotic treatment. Malignancies included both hematological or involving solid organs. Rejection grade were calculated as described by Stewart et al. [11].

Chronic kidney disease (CKD) was defined as stage 4 CKD according to an eGFR<30 mL/min/1.73mq, calculated through EPI-CKD equations.

### Statistical Analysis

Categorical variables were expressed as absolute frequency and percentage and quantitative variables as mean ± standard or median (interquartile range) according to data distribution, after performing the Kolmogorov-Smirnov test for normality.

Overall survival was estimated using the Kaplan–Meier method (log-rank test). Cox-regression model estimated factors independently associated with long-term mortality and grade CAV. A difference was considered statistically significant if *p* < 0.05. All statistics were performed using the Statistical Package for Social Sciences (SPSS) program (Chicago, IL, USA).

## Results

During the study period, 349 patients underwent HTx at our center. Baseline recipients’ data about the period before HTx are reported in Table 1. The mean age was 56 ± 11 years and 81% were men. The primary indication for HTx was dilated cardiomyopathy (DCM) in 48% of patients, followed by ischemic cardiomyopathy (ICM) in 28%, and other diseases in 23%. Smoking was present in 39% of patients, with 18% active smoker and 21% formers. Thirteen percent had chronic obstructive pulmonary disease (COPD), 32% had chronic renal failure and 16% atrial fibrillation at the time of surgery.

**TABLE 1:** Baseline recipients’ data.

N. patients	349
Mean Age, years	56 ± 11
Male/Female, n (%)	283 (81)/66 (19)
Aetiology	
DCM, n (%)	168 (48)
ICM, n (%)	97 (28)
Other, n (%)	79 (23)
Re-HTx, n (%)	19 (5)
Previous CCH, n (%)	127 (36)
Smoking, n (%)	137 (39)
Active smoker, n (%)	62 (18)
Former smoker, n (%)	75 (21)
COPD, n (%)	13 (4)
Chronic Renal Failure, n (%)	112 (32)
Atrial fibrillation, n (%)	57 (16)
PM/ICD, n (%)	110 (32)
MCS, n (%)	72 (21)
Median LVEF, %	27 (20–35)
Mean sPAP, mmHg	43 ± 1

CCH, cardiac surgery; COPD, chronic obstructive pulmonary disease; DCM, dilated cardiomyopathy; ICD, implantable cardioverter-defibrillator; ICM, ischemic cardiomyopathy; HTx, heart transplantation; LVEF, Left Ventricular Ejection Fraction; MCS, Mechanical Circulatory Support; PM, pacemaker; sPAP, systolic pulmonary artery pressure.

During a median follow-up of 53 (16–112) months, late mortality was 30%. Most common complications were infection episodes in 32% of patients, acute rejection grade ≥2 in 24%, malignancies in 19%, Cytomegalovirus (CMV) infection in 17%, renal failure grade ≥4 in 15% and CAV grade ≥2 in 9% (Table 2).

**TABLE 2:** Long-term outcome.

N. patients	332
Median follow-up, months	53 (16–112)
Late mortality, n (%)	100 (30%)
Acute rejection grade ≥ 2, n (%)	80 (24%)
Infection, n (%)	106 (32%)
CMV infection, n (%)	55 (17%)
Malignancies, n (%)	63 (19%)
CAV grade ≥ 2, n (%)	30 (9%)
Renal failure grade ≥ 4, n (%)	48 (15%)

CAV, cardiac allograft vasculopathy; CMV, Citomegalovirus.

**FIGURE 1:**
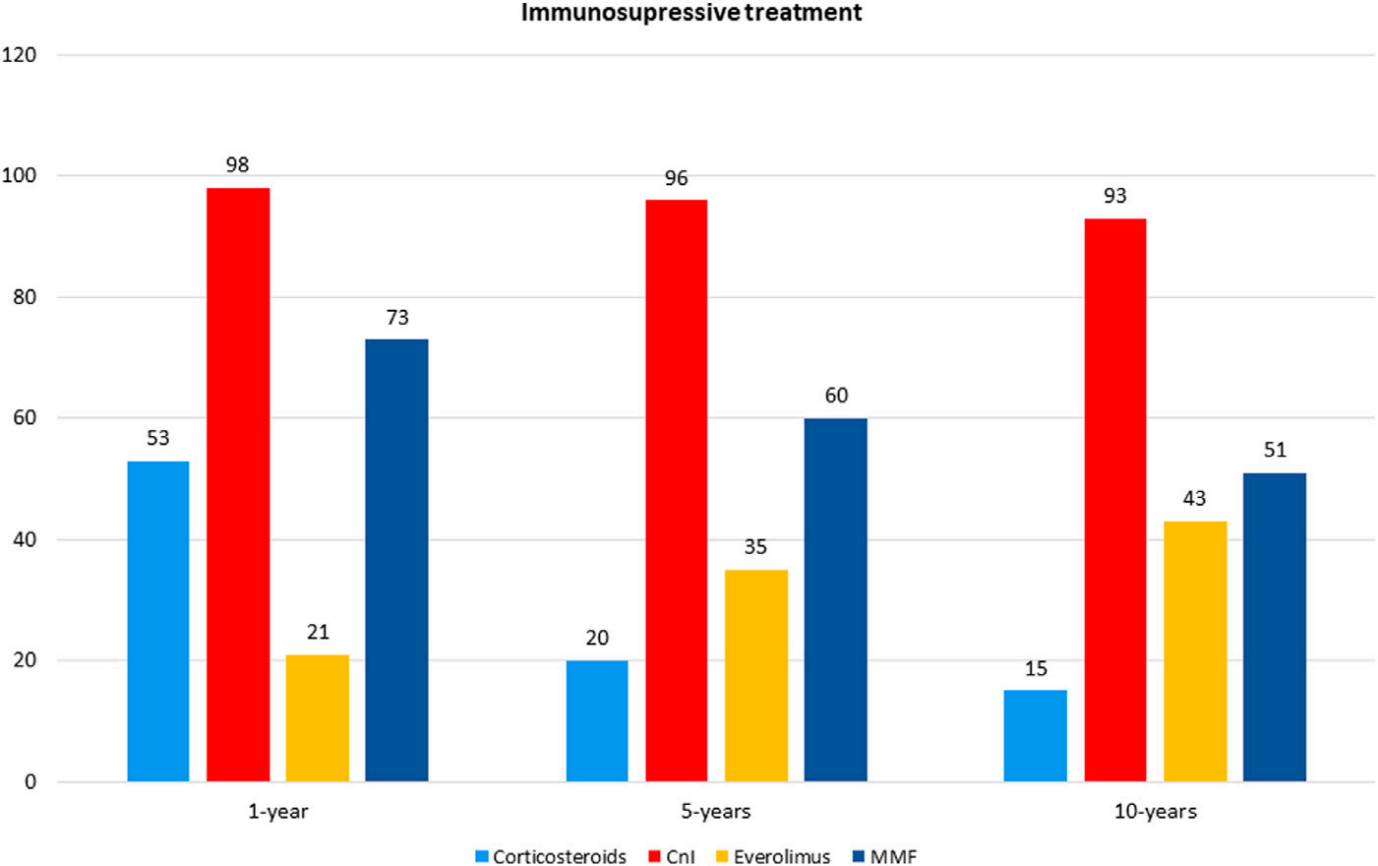
Immunosuppressive therapuy.

Figure 1 and Table 3 shows the patients’ immunosuppressive treatment during follow-up. At the first year after HTx the most frequent combination of immunosuppression medications was Cys + MMF with corticosteroids (31%) or without (29%). At 5 and 10 years of follow-up, the Cys + MMF combination therapy remained the most prescribed treatment (44% and 48% at 5 and 10 year follow-up, respectively), followed by a progressive increased in the Cys + Everolimus prescription (29% and 33% at 5 and 10 year follow-up, respectively). The use of corticosteroids decreased over the time, according to our center protocol, shifting from a 53% of patients at the first year after HTx, in combination with the other immunosuppressive drugs, to a 19% and a 15% at 5 and 10 years of follow-up, respectively.

**TABLE 3:** Immunosuppressive treatment during study period.

	1 year f-up	5 year f-up	10 year F-up
N. of patients	280	166	80
Cys + MMF + Corticosteroids	86 (31)	12 (7)	2 (3)
Cys + Eve + Corticosteroids	23 (8)	6 (4)	3 (4)
Cys + AZA + Corticosteroids	5 (2)	0	0
Tacrolimus + MMF + Corticosteroids	18 (6)	4 (2)	1 (1)
Tacrolimus + Everolimus + Corticosteroids	2 (0.7)	0	1 (1)
Cys + MMF	82 (29)	73 (44)	38 (48)
Cys + Everolimus	29 (10)	48 (29)	26 (33)
Cys + Corticosteroids	10 (4)	6 (4)	1 (1)
Tacrolimus + MMF	19 (7)	11 (6)	2 (3)
Tacrolimus + Corticosteroids	1 (0.4)	1 (0.6)	0
Tacrolimus + Everolimus	1 (0.4)	0	1 (1)
MMF + Corticosteroids	1 (0.4)	0	1 (1)
Everolimus + Corticosteroids	3 (1)	5 (3)	3 (4)
Cys + AZA	0	0	1 (1)

AZA, azathioprine; Cys, Cyclosporine; MMF, mycophenolate mofetil.

### Metabolic Syndrome Prevalence

As regard the prevalence of MetS, 35% of patients already satisfied the criteria for the diagnosis before HTx. During the follow-up, this percentage steadily grew, with 47% of patients at the first year after HTx, 52% at 5% and 46% at 10 years of follow-up. In particular, among the 131 patients with MetS at 1 year after HTx, only 60 (46%) had MetS before HTx too.

Focusing on the singular criteria, half of the patients (50%) had TGL ≥150 mg/dL or was prescribed with treatment for hypertriglyceridemia before HTx, while this number increased during the follow-up, with 92% of patients at 1 year of follow-up, 89% at 5 years and 93% at 10 years. Similarly, 34% of patients had hypertension (HTN) or took an anti-HTN treatment prior to HTx, but the percentage reached 86% at 1 year of follow-up, 90% at 5 years, and 91% at 10 years. As regard obesity, 12% of patients had a BMI >30 before HTx, while within the first year after HTx obese patients were 19%, 25% at 5 years and 20% at 10 years. DM and glucose blood level appeared, instead, to be halved: while 61% of patients had DM or fasting hyperglycemia pre-HTx, at 1, 5 and 10 years of follow-up the frequencies were respectively 35%, 43% and 38%. Finally, also low HDL blood level, presented in 34% of patients before HTx, resulted decreased during the follow-up, with 18% of patients at 1 year, 20% at 5 years and 16% at 10 years (Table 4).

**TABLE 4:** Prevalence of Metabolic syndrome before and after Heart Transplantation.

Prevalence of MetS	Pre-HTx	1 year f-up	5 year f-up	10 year F-up
N. of patients	349	280	166	80
TGL ≥150 mg/dL or hypertriglyceridemia drugs, n (%)	173 (50)	257 (92)	147 (89)	74 (93)
HDL <40 mg/dL in men or <50 mg/dL in women, n (%)	120 (34)	49 (18)	33 (20)	13 (16)
DM or glucose ≥100 mg/dL	211 (61)	97 (35)	72 (43)	30 (38)
Blood pressure ≥ 130/85 mmHg or HTN drugs, n (%)	120 (34)	242 (86)	149 (90)	73 (91)
BMI > 30, n (%)	43 (12)	53 (19)	41 (25)	16 (20)
MetS, n (%)	123 (35)	131 (47)	86 (52)	37 (46)

BMI, body mass index; DM, diabetes mellitus; HDL, high density lipoprotein cholesterol; HTN, hypertension; MetS, metabolic syndrome; TGL, triglyceride.

### Mortality and Morbidity Predictors

**FIGURE 2:**
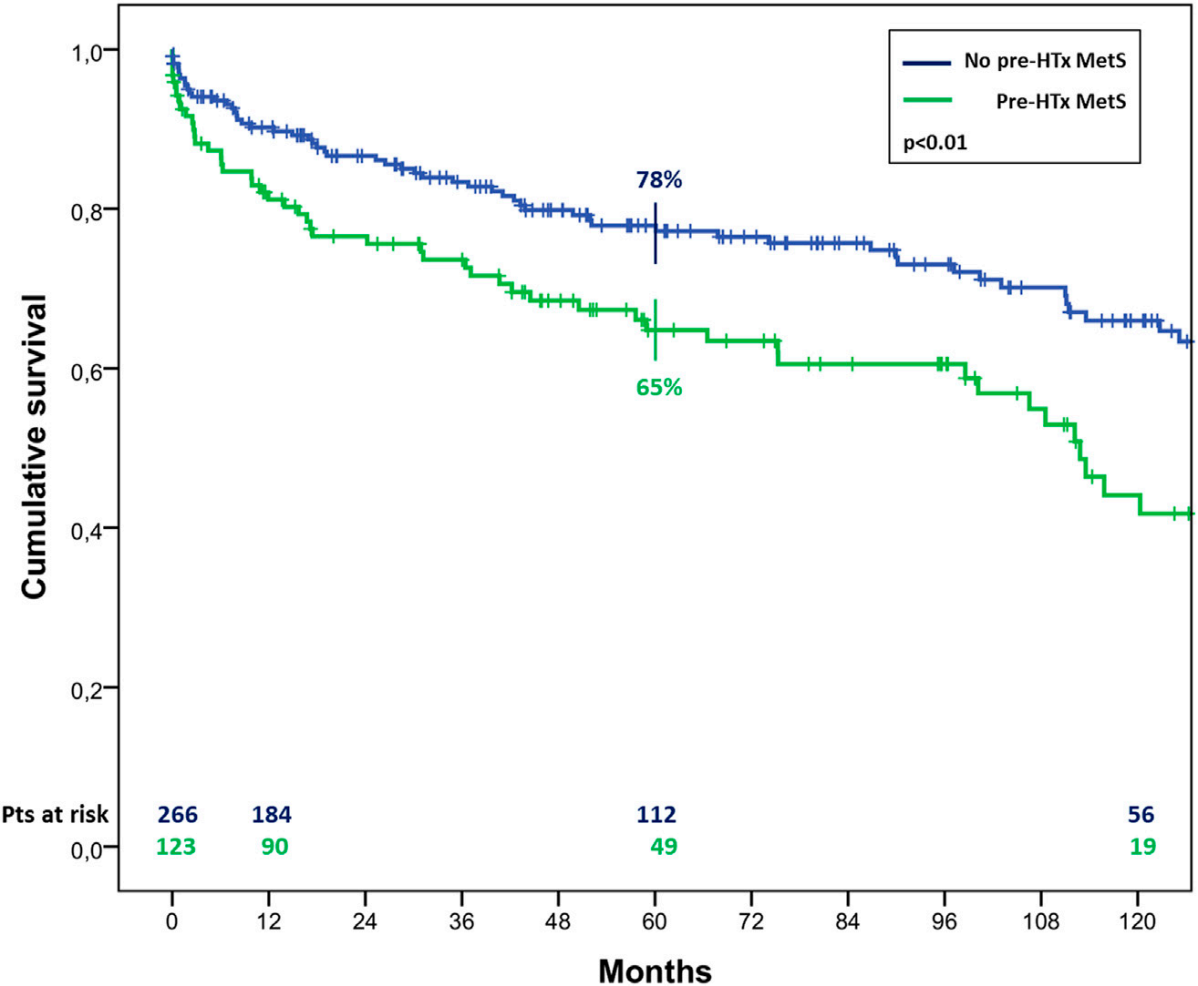
Cumulative survival in cardiac transplanted patients with or without MetS before HTx.

**FIGURE 3:**
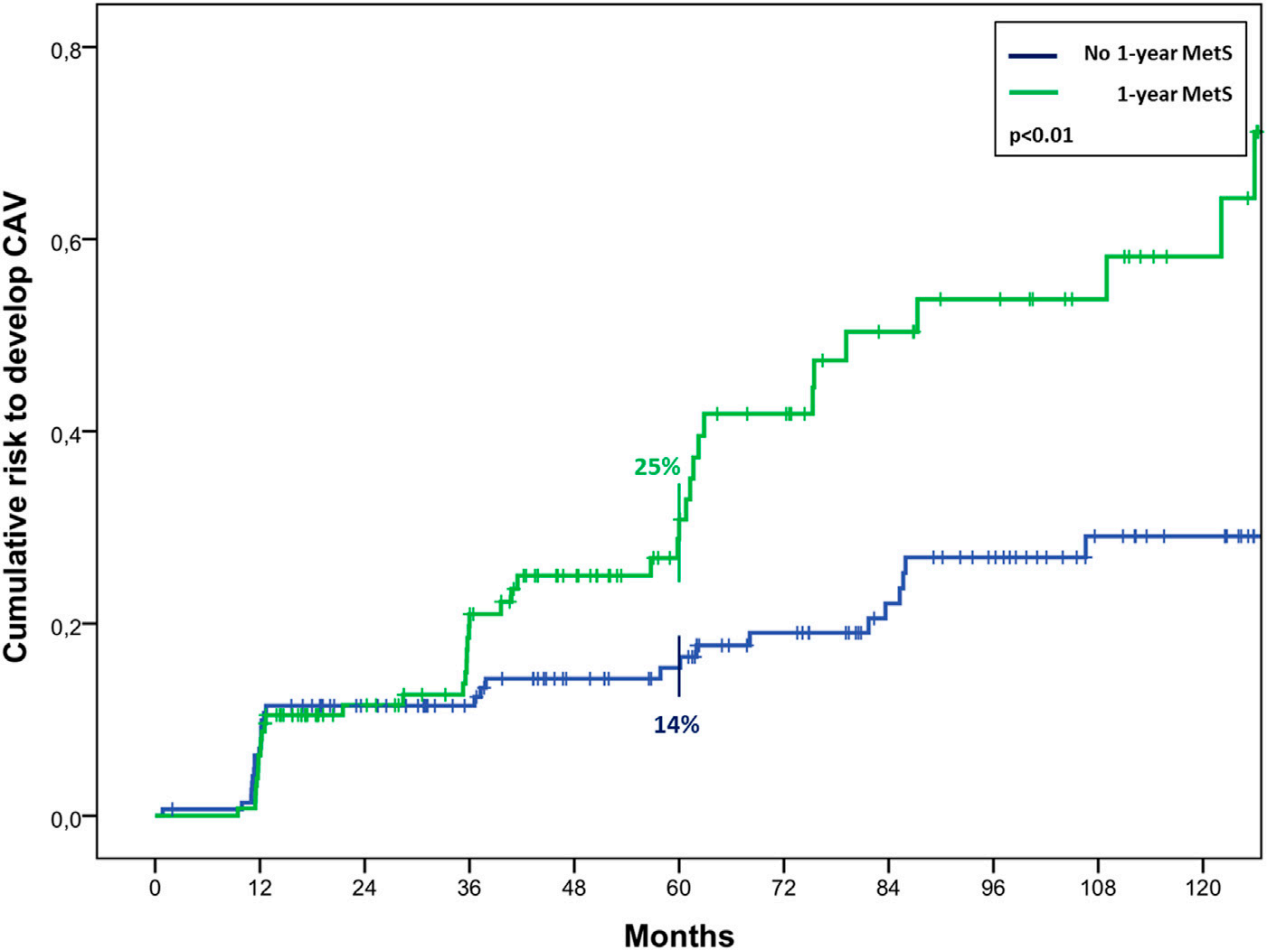
Cumulative survival in cardiac transplanted patients with or without MetS at 1 year after HTx.

The overall survival in patients with MetS before HTx appeared significantly worst, resulting of 81% ± 4% vs. 90% ± 2%, 65% ± 5% vs. 78% ± 3% and 44% ± 6% vs. 66% ± 4% (*p* < 0.01) at 1, 5, and 10 years of follow up in patients with and without pre-HTx MetS, respectively (Figure 2). Similar results were found also in patients with MetS at the first year of follow-up, with a survival of 78% ± 4% vs. 89% ± 3% and 57% ± 6% vs. 75% ± 5% (*p* < 0.01) at 5 and 10 years of follow up in patients with and without MetS at 1 year follow-up, respectively (Figure 3).

**FIGURE 4:**
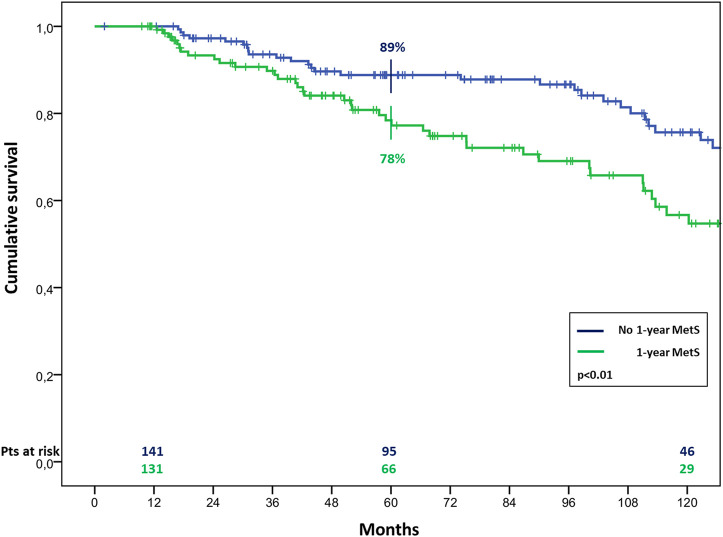
Cumulative risk to develop CAV in cardiac transplanted patients with or without MetS 1 year after HTx.

At the univariate analysis, risk factors for mortality were recipient age (HR 1.07, 1.04–1.09, *p* < 0.01), pre-HTx MetS (HR 1.86, 1.29–2.69, *p* < 0.01), pre-HTx HTN (HR 2.46, 1.70–3.55, *p* < 0.01), pre-HTx hypertriglyceridemia (HR 1.50, 1.04–2.18, *p* = 0.03), chronic renal failure (HR 2.95, 2.03–4.27, *p* < 0.01), MetS and DM at 1 year follow-up (HR 2.00, 1.25–3.19, *p* < 0.01; HR 2.02, 1.27–3.23, *p* < 0.01, respectively). The last two resulted also risk factors for CAV (HR 1.86, 1.16–2.99, *p* = 0.01; HR 1.67, 1.03–2.69, *p* = 0.04, respectively). In particular, MetS at 1-year follow-up determined a significant higher risk to develop CAV, resulting in a risk of 25% ± 4% vs. 14% ± 3% at 5 years after HTx, and 44% ± 6% vs. 25% ± 4% at 10 years (*p* < 0.01) (Figure 4).

## Discussion

The main findings of this study were a) MetS was highly prevalent in HTx patients of our center, with hypertriglyceridemia and hypertension being the most common increased metabolic factors; b) both MetS before and at 1 year after HTx determined a significative worst survival, resulting also as risk factors for mortality at the univariate analysis; c) MetS at 1 year after HTx determined a significant higher risk to develop CAV.

MetS is a multi-factorial condition, a cluster of metabolic risk factors (abdominal obesity, dyslipidemia, high blood glucose, high blood pressure), frequently observed in clinical practice, especially after HTx. In these patients, in fact, MetS represents a burden that strongly affect their long-term outcome, mainly correlated to the side effects of the lifelong immunosuppressive therapy.

In this study, we firstly focused on analyzing the prevalence and the evolution of MetS in HTx patients of our center over 10 years of follow-up. Only a limited number of studies, to date, have discussed this topic, considering different timepoints. Martinez-Dolz et al., for instance, evaluated the prevalence of early MetS (pre-HTx or in the first 3 months post-HTx), which resulted to be 41.9%, in line with other studies concerning liver or renal transplantation [8, 12, 13]. A similar percentage was also found by Cordero et al., who reported a 43% prevalence of MetS in 111 HTx patients after 8 ± 6 years from transplant [2]. However, the prevalence reported in our study was even higher, with 47% of patients being affected by this condition at 1 year after HTx and more than half of them (52%) at 5 years of follow-up. The prevalence seemed to increase over the follow-up period, suggesting a possible association with greater exposure time to immunosuppressive treatment.

Analyzing the parameters involved in the MetS diagnosis, it was observed a surge in the hypertension/anti-hypertensive treatment criterion and in the hypertriglyceridemia/treatment for hypertriglyceridemia criterion over the three timepoints considered, as well as an increase in BMI. Several studies have shown an attitude to the development of hypertension, dyslipidemia and obesity in transplant population during the follow-up, mostly correlated to the side effects of immunosuppressive therapy [14–16]. Data from the International Society for Heart and Lung Transplantation (ISHLT) showed that hypertension is present in 50%–90% of transplant patients and is associated with increased cardiovascular morbidity and mortality [17]. It has been shown that patients receiving cyclosporine develop new-onset hypertension requiring pharmacological treatment in 82% of cases [15]. Other metabolic side effects related to cyclosporine use are hyperlipidemia and *de novo* diabetes mellitus at 1 year, which is present as many as 10% of patients, as long as a higher risk to develop osteoporosis [14]. Weight gain and obesity, instead, are mainly correlated with the use of glucocorticoids [18], with an approximately 10 kg gain, on average, in the first year after HTx [16].

As regard results about mortality, the patients in this series who met early MetS criteria, showed a significantly worst long-term survival, with a 5 year survival of 65% vs. 78% for patients with or without MetS before HTx, and 78% vs. 89% for patients with or without MetS at 1 year after HTx, respectively. These results are a confirmation of the hypothesis stated by Martinez-Dolz et al., according to whom the chronological development of MetS is a relevant concern regarding its prognostic value [8]. Interestingly, among the MetS criteria analyzed, three were found to be independent risk factors for mortality: HTN and hypertriglyceridemia prior to HTx and DM at 1 year follow-up. The latest report of the ISHLT registry identified the recipient history of diabetes as an independent risk factor for mortality after both 5 and 10 years after HTx [17]. In particular, new onset DM after HTx has been reported to be associated to an increased risk of cardiovascular incidents resulting in death and other diseases. Other adverse effects included infection, rejection, and early graft loss [19].

In HTx patients, different metabolic abnormalities have been associated with the development of CAV or chronic rejection, which is one of the main causes of graft failure and death over the long-term follow-up after HTx [5, 20] CAV is considered a rapid form of atherosclerosis confined to the graft, caused by an endothelial dysfunction of multifactorial origin. Since MetS is characterized by a chronic systemic inflammation which induces endothelial dysfunction [21], it is reasonable to expect an impact of MetS on the development of CAV. Indeed, in this study the univariate analysis showed MetS and DM at 1 year after HTx to be associated to the development of CAV. A similar association was also found by Sanchez-Gomez et al., where 67% of patients with MetS developed CAV, being the presence of MetS an independent predictor with an OR of 7.97 [4]. At the univariate analysis, they found the MetS components hypertriglyceridemia, high BMI and low HDL-C levels to be associated with the CAV. In our study only DM resulted associated, but considering that insulin resistance (IR) is a known cause of endothelial cells dysfunction and one of the main player involved in triggering MetS [21], a consequent correlation between all the other associated metabolic components appears clear. Moreover, an association between IR and CAV was already been described in a study by Valantine et al [22]. They showed that metabolic markers of IR are significantly correlated with coronary artery intimal thickening in the transplanted heart subjects and that this metabolic abnormality significantly predicted the development of CAV and death during the subsequent 5 years of follow-up. Another interesting confirmation comes from a prospective, cross-sectional study by Raichlin et al, in which, evaluating blood samples from HTx patient on average nearly 5 years after transplantation, markers of IR and systemic inflammation independently identified patients at higher risk for subsequent angiographic CAV and cardiovascular events [23].

All these results underline the importance to keep monitored the metabolic alterations after HTx, during the follow-up of the patients. Patients with MetS diagnosis before or within the first year of HTx should be followed closer, as they are more prone to develop cardiovascular events. Indeed, immunosuppressive therapy plays a primary role in the development and progression of MetS and the associated components. However, also inadequate dietary habits and physical inactivity might strongly affect the metabolic status of these patients and could represent two important tools to monitor the onset or evolution of this multifactorial condition. Adding a nutritional support and a physical activity program in the standard follow-up care of HTx patients might outline a valid strategy to limit the prevalence of MetS.

This study has some limitations related to its single-center retrospective nature and the results may not be as representative as multi-center reports, but may add valuable data in a topic, as MetS after HTx, not frequently described in literature. Moreover, concerning a specific pathological population, the sample size resulted automatically limited compared to other studies about MetS in general population. Another limitation was the failure to establish the actual contribution of immunosuppressive therapy on the development of the factors associated with MetS, because of its gradually modification over time and among patients during the study period. Further analysis, possibly through a multi-center study, are needed to better explore the impact and features of MetS after HTx.

In conclusion, this study confirmed the high prevalence of MetS in the sample of HTx patients of our center, and the presence of early MetS, both before and at 1 year after HTx, resulted in a significant worst outcome in terms of survival and development of CAV.

## Data Availability

The raw data supporting the conclusion of this article will be made available by the authors, without undue reservation.

## Abbreviations

BMI, Body Mass Index; CAV, Coronary Allograft Vasculopathy; CKD, Chronic Kidney Disease; CMV, Citomegalovirus; COPD, Chronic Obstructive Pulmonary Disease; Cys, Cyclosporine; DCM, Dilated Cardiomyopathy; DM, Diabetes Mellitus; HDL, High-Density Lipoprotein; HTN, Hypertension; HTx, Heart Transplantation; ICM, Ischemic Cardiomyopathy; MetS, Metabolic Syndrome; MMF, Mycophenolate Mofetil; TGL, Triglyceride.
